# Association between self-initiated changes in physical activity patterns and erectile dysfunction: a longitudinal follow-up study

**DOI:** 10.31744/einstein_journal/2026AO1905

**Published:** 2026-06-23

**Authors:** Rafael Mathias Pitta, Oskar Grau Kaufmann, Eduardo Rossato de Victo, Gabriel Grizzo Cucato, Raphael Mendes Ritti-Dias, Luana de Lima Queiroga, Nelson Wolosker

**Affiliations:** 1 Hospital Israelita Albert Einstein Faculdade Israelita de Ciências da Saúde Albert Einstein São Paulo SP Brazil Postgraduate Program in Health Sciences, Faculdade Israelita de Ciências da Saúde Albert Einstein, Hospital Israelita Albert Einstein, São Paulo, SP, Brazil.; 2 Northumbria University Sport, Exercise & Rehabilitation Northumbria Newcastle United Kingdom Sport, Exercise & Rehabilitation, Northumbria University, Northumbria, Newcastle, United Kingdom.; 3 Universidade Nove de Julho Postgraduate Program in Health Sciences São Paulo SP Brazil Postgraduate Program in Health Sciences, Universidade Nove de Julho, São Paulo, SP, Brazil.

**Keywords:** Erectile dysfunction, Exercise, Sexual behavior

## Abstract

**Objective:**

The effect of self-initiated changes in physical activity patterns over time on erectile dysfunction remains unclear. We aimed to analyze the relationship between self-initiated changes in physical activity over time and erectile dysfunction in a large sample of men.

**Methods:**

This study included 925 adults and older men with erectile dysfunction who underwent routine health screening. Erectile dysfunction was determined using the 5-item version of the International Index of Erectile Function questionnaire, with a score of <21 indicating the condition. The participant's physical activity levels, body mass index, blood pressure, blood samples, alcohol consumption, tobacco use, perceived stress, depressive symptoms, and lower urinary tract symptoms were measured at baseline and follow-up (531,9±149,7 days). The participants were grouped into four categories based on their physical activity levels at baseline and follow-up: i) persistently physically inactive, ii) became physically inactive, iii) became physically active, and iv) persistently physically active.

**Results:**

Hierarchical regression analysis revealed that individuals who became physically active and those who remained physically active were independently associated with decreased odds of erectile dysfunction (odds ratio [OR], 0.656; 95%CI=0.432-0.980 and OR=0.717; 95%CI=0.508-0.992, respectively).

**Conclusion:**

Self-initiated changes in physical activity patterns were longitudinally associated with erectile dysfunction in adults and older individuals. These data suggest that initiating and maintaining physical activity can reduce the risk of erectile dysfunction.

## In Brief

Self-initiated increases and maintenance of physical activity over time were associated with a low risk of erectile dysfunction in adults and older men. In a longitudinal analysis of 925 participants, men who became physically active or remained physically active showed significantly reduced odds of erectile dysfunction compared with persistently inactive individuals. These findings suggest that adopting and sustaining an active lifestyle may play a protective role against erectile dysfunction.

## Highlights

■ Becoming physically active was associated with low odds of erectile dysfunction.
■ Remaining physically active was associated with low odds of erectile dysfunction.

## Graphical abstract

**Figure:**
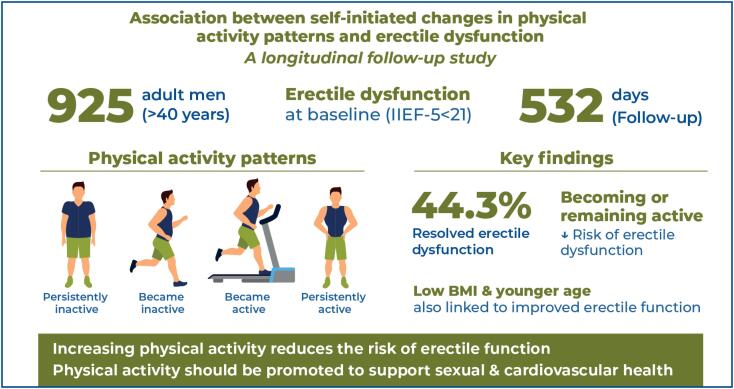

## INTRODUCTION

Erectile dysfunction (ED) is linked to cardiovascular risk factors. It is clinically defined as the inability to achieve and/or maintain sufficient penile erection for satisfactory sexual intercourse.^(1,2)^ The prevalence and incidence of ED increase with age and can reach up to 70% in older adults.^(2,3)^ This condition has been associated with impaired quality of life in men.^(2)^

Among protective factors, modifiable lifestyle changes are known to be associated with ED.^(2)^ In cross-sectional studies, physical activity (PA) has been consistently negatively correlated with ED.^(2,4,5)^ However, real-world cross-sectional population studies on this association^(4)^ have only focused on exercise in the leisure-time domain, and these data are limited to developed countries. Additionally, retrospective analysis^(2,5)^ does not enable us to determine the effect of changes in PA patterns over time on ED.

Successful execution of these intervention programs in real-life public health settings poses challenges.^(6)^ Previous studies^(6,7)^ have shown that self-reported changes in PA patterns are linked to improvements in various clinical and health indicators in Brazilian adults. However, the potential long-term impact of informal and self-initiated PA in everyday conditions on these indicators is not yet understood.

The relationship between PA patterns and ED in middle-income countries is poorly understood.^(2,5)^ Although cross-sectional studies have shown clear associations between PA and ED,^(2,4,5)^ the effect of self-initiated changes in PA patterns over time on ED remains unclear. Given that ED is an important marker of cardiovascular risk, low-grade inflammation, and quality of life in men, it would be valuable to explore in a follow-up study whether individuals who maintain or increase their PA levels experience ED.

## OBJECTIVE

We aimed to analyze the association between changes in physical activity patterns over time (persistent inactivity, becoming inactive, becoming active, and persistent activity) and the incidence of erectile dysfunction in in a large population (925 Brazilian men). We hypothesized that individuals who become or remain physically active over time have a low risk of developing erectile dysfunction.

## METHODS

In this longitudinal cohort study, we analyzed PA and ED at baseline and follow-up visits in 925 men. The time between visits was 300 and 800 days after the initial assessment. The sample consisted of Brazilian men aged >39 years who participated in an employer-sponsored routine health evaluation at the Preventive Medicine Center of a quaternary hospital in São Paulo between January 2008 and December 2022. All procedures were approved by the Ethics Committee of *Hospital Israelita Albert Einstein* (SGPP: 592924) in compliance with the Brazilian National Research Ethics System Guidelines (CAAE: 77752224.0.0000.0071; # 6.753.268). A waiver of informed consent was requested and granted owing to the nature of this study, which did not add risks or prejudice to the well-being of the patients. Additionally, the waiver of the Free and Informed Consent Form was justified by the lack of regular follow-up visits. Patient data confidentiality was guaranteed owing to strict internal protocols.

All participants were invited to participate voluntarily in the health promotion and prevention programs. The initial database consisted of 125,439 adult men and women who underwent health checkups. This study included only individuals who returned for follow-up visits between 300 and 800 days, as defined in previous studies.^(6,7)^ We excluded female checkups, duplicate data, missing data on PA and ED, individuals aged <40 years who reported no sexual activity in the previous year (assessed with a yes or no question), and those with penile prostheses. Individuals who had undergone more than two health screenings were also excluded, with the last visit being used as the follow-up. We further excluded individuals who did not show some degree of ED at baseline (as indicated by International Index of Erectile Function results of >21 points). Finally, data on PA and ED from 925 males, with a mean time between visits of 531,9±149,7 days, were analyzed.

### Clinical data

The body mass index (BMI) was calculated using the formula weight/height^2^. Weight was measured using the InBody 230 equipment (Ottoboni^®^), and height was measured using a stadiometer.

Blood samples were collected after overnight fasting and analyzed using a routine clinical workflow. Laboratory analyses included measurements of glycated hemoglobin (HbA1C, %), lipid profile (mg/dL), uric acid (UA,mg/dL), and ultrasensitive C-reactive protein (CRP,mg/dL) using a Vitros platform automated laboratory system (Johnson & Johnson Clinical Diagnostics, New Brunswick). The laboratory responsible for all blood analyses met the standardized criteria for quality control adopted by the Brazilian Health Ministry. C-reactive protein levels were measured using the turbidimetric method on a nephelometry system (Dade Behring, USA).

Blood pressure was measured in triplicate while the participants were seated and rested for at least 5 min, following the American Heart Association Guidelines.^(8)^ Measurements were performed on both arms using the auscultatory method with an aneroid sphygmomanometer and phases I and V of the Korotkoff sounds.

Medical records were reviewed to determine the presence of comorbidities, such as systemic arterial hypertension, diabetes mellitus, dyslipidemia, nonalcoholic fatty liver steatosis, smoking status, and medication use. If the necessary data were not available for medical assessment, we considered the presence of hypertension and diabetes based on self-reported information from the patients, including chronic use of antihypertensive medications, self-reported diabetes mellitus, or self-reported chronic use of anti-diabetic medications. Lower urinary tract symptoms were assessed through face-to-face interviews using the International Prostatism Symptom Score.^(9)^

Lifestyle factors were assessed using standardized questionnaires that were administered by trained professionals. The Perceived Stress Scale^(10)^ was used to assess perceived stress. Psychologists used the Beck Depression Inventory-II^(11)^ to determine the presence and level of depression. Alcohol consumption was evaluated using the Alcohol Consumption Disorders Identification Test.^(12)^

### Physical activity

PA levels were measured using the International Physical Activity Questionnaire.^(13)^ The International Physical Activity Questionnaire assesses the volume (time spent), intensity (light, moderate, and vigorous), and sedentary activity in a typical week. The patients were classified into four groups: highly active, active, low active, and physically inactive. Participants who engaged in at least 30 min of vigorous PA on at least 5 days per week or those who engaged in at least 20 min of vigorous PA on at least 3 days per week and were associated with moderate PA and/or walking for at least 30 min on at least 5 days per week were classified as highly active. Individuals who performed at least 20 min of vigorous PA on at least 3 days a week or those who performed any PA for at least 150 min a week over at least 5 days were considered active. Individuals who reported engaging in PA but did not meet the above criterion (150 min/week) were classified as low active. Those who reported no PA were considered inactive. Thus, the highly active and active groups were considered physically active, whereas the low active and inactive groups were considered physically inactive.

Finally, for the longitudinal assessment of changes in PA patterns between baseline and follow-up visits, four groups were created: i) persistently physically inactive (inactive at both baseline and follow-up), ii) became physically inactive (active at baseline and inactive at follow-up), iii) became physically active (inactive at baseline and active at follow-up), and iv) persistently physically active (active at both baseline and follow-up).

### Erectile dysfunction

The presence and severity of ED were assessed using the 5-item version of the International Index of Erectile Function.^(14)^ The questionnaire contains five questions covering two domains: erectile function and intercourse satisfaction. Respondents rated their answers on a scale of 1 to 5. The total score ranged from 5 to 25, with low scores indicating poor sexual function. ED severity was classified into five categories based on the scores: severe (5-7), moderate (8-11), mild-to-moderate (12-16), mild (17-21), and no ED (22-25). Based on the results, the presence of ED was identified in individuals with a score of ≤21, including severe, moderate, mild-to-moderate, and mild categories.

All patients were evaluated at baseline and follow-up appointments to assess the variables. Doctors and a multidisciplinary team (nutritionists, physical education professionals, psychologists, physiotherapists, and nurses) advised patients with clinical and behavioral changes to modify their lifestyles to improve their health. Patients with any level of ED were advised to start or maintain professional follow-ups. Initially, we compared the demographic, clinical, and lifestyle variables at baseline with respect to changes in PA patterns. Subsequently, we compared the demographic, clinical, and lifestyle variables at baseline with respect to ED. Finally, we assessed the association between all factors studied in ED, focusing on the association between changes in PA patterns and ED.

### Data analysis

We used the Shapiro-Wilk test to assess data normality. Categorical variables are presented as frequencies and percentages, whereas descriptive statistics for continuous variables are presented as means and standard deviations. Categorical variables were compared using the chi-square and likelihood ratio tests. When comparing numerical variables concerning changes in PA patterns, we used the one-way analysis of variance and Bonferroni tests for multiple comparisons. Student's *t*-test was used to compare numerical variables related to ED.

We measured independent variables at two time points (baseline and follow-up) and created variables using a longitudinal approach. The association between changes in PA patterns and ED was estimated using hierarchical regression and expressed as odds ratios (OR) and 95% confidence intervals (95%CIs).

The baseline values of age, BMI, presence of hypertension, presence of diabetes, tobacco use, presence of dyslipidemia, alcohol consumption (measured using the Alcohol Consumption Disorders Identification Test), perceived stress (assessed using the Perceived Stress Scale), depressive symptoms (measured using the Beck Depression Inventory-II), lower urinary tract symptoms (evaluated using the International Prostatism Symptom Score), and changes in PA patterns were used as covariates in the hierarchical models. We adjusted the models in three steps: first, by age (each year) and BMI (kg/m²); then, by adding hypertension, diabetes, dyslipidemia, and smoking status; finally, we added alcohol consumption, perceived stress, depressive symptoms, lower urinary tract symptoms, and changes in PA patterns. A p<0.05 was considered significant. All statistical analyses were performed using SPSS for Windows (version 24.0; IBM Corp., Armonk, NY, USA).

## RESULTS

The study sample included 925 males with ED at baseline. During the follow-up, 410 (44.3%) participants no longer reported ED, 296 (32%) had mild ED, 23 (2.5%) had mild-to-moderate ED, 148 (16%) had moderate ED, and 48 (5.2%) had severe ED. In addition, 283 (30.6%) participants remained physically inactive, 130 (14.1%) became physically inactive, 158 (17.1%) became physically active, and 354 (38.3%) remained physically active. The demographic, clinical, and behavioral data related to changes in PA patterns at baseline are presented in table 1. Persistently physically inactive individuals had a higher BMI (29±4.4 *versus* 27.1±3.6kg/m², p<0.001) than persistently physically active individuals. Those who became physically inactive also had a higher BMI (28.2±3.4 *versus* 27.1±3.6kg/m², p<0.001) than persistently physically active individuals. Furthermore, individuals who became physically active individuals had a higher BMI (28.1±3.9 *versus* 27.1±3.6kg/m², p<0.001) than persistently physically active individuals.

**Table 1:** Comparison of demographic, clinical, and lifestyle data at baseline in relation to changes in physical activity patterns (n=925)

		Persistently physically inactive	Became physically inactive	Became physically active	Persistently physically active	Total	p value
Age (years) mean±SD (n)		49.9±7.8 (283)	50.8±8.7 (130)	50.8±8.2 (158)	51.1±9.1 (354)	50.6 ±8.4 (925)	0.312
BMI (kg/m²) mean±SD (n)		29±4.4 (283)§	28.2±3.4 (130)&	28.1±3.9 (158)¥	27.1±3.6 (354)	28±4 (925)	<0.001*
Glycated hemoglobin (%) mean±SD (n)		5.7±1.0 (240)§	5.5±0.9 (117)	5.6±0.9 (142)	5.5±0.7 (307)	5.6±0.9 (806)	0.074
Total cholesterol (mg/dL) mean±SD (n)		195±37.7 (283)	185.1±41.2 (130)	191.6±36.9 (158)	187.8±37.2 (353)	190.3±38 (924)	0.035*
LDL cholesterol (mg/dL) mean±SD (n)		122.6±33.2 (283)	113.7±36.9 (130)	120.5±32 (158)	115.6±33.6 (353)	118.3±33.8 (924)	0.018*
HDL cholesterol (mg/dL) mean±SD (n)		42.9±9.2 (283)§	44±10.4 (130)&	44.4±11.4 (158)¥	48.2±10.9 (353)	45.4±10.7 (924)	<0.001*
Triglycerides (mg/dL) mean±SD (n)		160.7±101.2 (283)§	154.1±125.9 (130)&	146.2±78.7 (158)	124.4±64.5 (353)	143.4±90.7 (924)	<0.001*
Uric acid (mg/dL) mean±SD (n)		6.2±1.3 (276)‡ §	6.0±1.1 (130)	5.9±1.1 (154)	5.9±1.2 (349)	6±1.2 (909)	0.013*
U-RCP (mg/L) mean±SD (n)		0.3±0.6 (109)	0.2±0.2 (40)	0.6±2.3 (69)	0.2±0.4 (118)	0.3±1.1 (336)	0.115
Hypertension n (%)							
	Absence	201 (71)	87 (66.9)	116 (73.4)	251 (70.9)	655 (70.8)	0.687
	Presence	82 (29)	43 (33.1)	42 (26.6)	103 (29.1)	270 (29.2)	
	Total	283 (100)	130 (100)	158 (100)	354 (100)	925 (100)	
Diabetes n (%)							
	Absence	249 (88)	119 (91.5)	143 (90.5)	323 (91.2)	834 (90.2)	0.517
	Presence	34 (12)	11 (8.5)	15 (9.5)	31 (8.8)	91 (9.8)	
	Total	283 (100)	130 (100)	158 (100)	354 (100)	925 (100)	
Dyslipidemia n (%)							
	Absence	139 (49.1)	61 (46.9)	78 (49.4)	159 (44.9)	437 (47.2)	0.692
	Presence	144 (50.9)	69 (53.1)	80 (50.6)	195 (55.1)	488 (52.8)	
	Total	283 (100)	130 (100)	158 (100)	354 (100)	925 (100)	
Tabagism status n (%)							
	Absence	256 (91.1)	120 (93)	146 (93)	323 (91.8)	845 (91.9)	0.868
	Presence	25 (8.9)	9 (7)	11 (7)	29 (8.2)	74 (8.1)	
	Total	283 (100)	129 (100)	157 (100)	352 (100)	919 (100)	
Perceived stress n (%)							
	Absence	183 (70.7)	81 (65.9)	99 (68.8)	262 (82.1)	625 (74)	<0.001†
	Presence	76 (29.3)	42 (34.1)	45 (31.3)	57 (17.9)	220 (26)	
	Total	259 (100)	123 (100)	144 (100)	319 (100)	845 (100)	
Alcohol consumption n (%)							
	Low-risk	246 (87.5)	109 (83.8)	130 (83.3)	291 (83.6)	776 (84.8)	0.733#
	Hazardous	30 (10.7)	19 (14.6)	22 (14.1)	52 (14.9)	123 (13.4)	
	Moderate-severe	5 (1.8)	2 (1.5)	4 (2.6)	5 (1.4)	16 (1.7)	
	Total	281 (100)	130 (100)	156 (100)	348 (100)	915 (100)	
Depressive symptoms n (%)							
	Minimum	228 (82.3)	94 (72.3)	136 (87.2)	324 (92.8)	782 (85.7)	<0.001#
	Mild	26 (9.4)	22 (16.9)	13 (8.3)	15 (4.3)	76 (8.3)	
	Moderate	20 (7.2)	11 (8.5)	5 (3.2)	9 (2.6)	45 (4.9)	
	Severe	3 (1.1)	3 (2.3)	2 (1.3)	1 (0.3)	9 (1)	
	Total	277 (100)	130 (100)	156 (100)	349 (100)	912 (100)	
Lower urinary tract symptoms n (%)							
	Absence/mild	234 (82.7)	111 (85.4)	140 (88.6)	303 (86.6)	788 (85.6)	0.690#
	Moderate	46 (16.3)	17 (13.1)	17 (10.8)	43 (12.3)	123 (13.4)	
	Severe	3 (1.1)	2 (1.5)	1 (0.6)	4 (1.1)	10 (1.1)	
	Total	283 (100)	130 (100)	158 (100)	350 (100)	921 (100)	

* ANOVA and multiple-comparison using Bonferroni test

† χ^2^ test;

# likelihood ratio.

a Persistently inactive *versus* Became inactive;

‡ Persistently inactive *versus* Became active;

§ Persistently inactive *versus* Persistently active;

d Became inactive *versus* Became active;

& Became inactive *versus* Persistently active;

¥ Became active *versus* Persistently active.

SD: standard deviation; BMI: body mass index; HDL: high-density lipoprotein; LDL: low-density lipoprotein; U-RCP: Urinary C-Reactive Protein.

In relation to clinical data, individuals who were persistently physically inactive had higher HbA1C (5.7±1.0 *versus* 5.5±0.7%, p<0.001), triglyceride (160.7±101.2 *versus* 124.4±64.5mg/dL, p<0.001), and UA (6.2±1.3 *versus* 5.9±1.2mg/dL, p<0.001) levels than persistently physically active individuals. Additionally, persistently physically inactive individuals had lower high-density lipoprotein cholesterol levels (42.9±9.2 *versus* 48.2±10.9mg/dL) than persistently physically active individuals. Regarding behavioral data, individuals who transitioned to being physically inactive exhibited higher perceived stress (p<0.001) and levels of depression (p<0.001).

The comparison of the demographic, clinical, and behavioral data related to ED is presented in table 2. Individuals with ED at follow-up were older (52.5±8.8 *versus* 48.3±7.3 years, p<0.001) and had higher BMI (28.4±4.1 *versus* 27.5±3.8 kg/m², p<0.001) and HbA1C levels (5.6±1 *versus* 5.5±0.6, p=0.007) than those without ED. They also had a higher incidence of hypertension (p<0.001), diabetes (p<0.001), dyslipidemia (p=0.022), and lower urinary tract symptoms (p=0.018) than individuals without ED.

**Table 2:** Comparison of demographic, clinical, and lifestyle data at baseline in relation to erectile dysfunction (n=925)

		Erectile dysfunction			p value
		Absence	Presence	Total	
Age (years) mean±SD (n)		48.3±7.3 (410)	52.5±8.8 (515)	50.6±8.4 (925)	<0.001*
BMI (kg/m²) mean±SD (n)		27.5±3.8 (410)	28.4±4.1 (515)	28±4 (925)	0.001*
Glycated hemoglobin (%) mean±SD (n)		5.5±0.6 (340)	5.6±1 (466)	5.6±0.9 (806)	0.007*
Total cholesterol (mg/dL) mean±SD (n)		192.5±36.8 (410)	188.5±38.9 (514)	190.3±38 (924)	0.109
LDL cholesterol (mg/dL) mean±SD (n)		120.7±33.2 (410)	116.4±34.2 (514)	118.3±33.8 (924)	0.052
HDL cholesterol (mg/dL) mean±SD (n)		46±10.9 (410)	44.8±10.5 (514)	45.4±10.7 (924)	0.091
Triglycerides (mg/dL) mean±SD (n)		137.3±76.4 (410)	148.3±100.5 (514)	143.4±90.7 (924)	0.069
Uric acid (mg/dL) mean±SD (n)		6±1.1 (404)	6±1.2 (505)	6±1.2 (909)	0.923
U-RCP (mg/L) mean±SD (n)		0.2±0.5 (180)	0.4 ±1.5 (156)	0.3±1.1 (336)	0.191
Hypertension n (%)					<0.001†
	Absence	317 (77.3)	338 (65.6)	655 (70.8)	
	Presence	93 (22.7)	177 (34.4)	270 (29.2)	
	Total	410 (100)	515 (100)	925 (100)	
Diabetes n (%)					<0.001†
	Absence	390 (95.1)	444 (86.2)	834 (90.2)	
	Presence	20 (4.9)	71 (13.8)	91 (9.8)	
	Total	410 (100)	515 (100)	925 (100)	
Dyslipidemia n (%)					0.022†
	Absence	211 (51.5)	226 (43.9)	437 (47.2)	
	Presence	199 (48.5)	289 (56.1)	488 (52.8)	
	Total	410 (100)	515 (100)	925 (100)	
Tabagism status n (%)					0.431
	Absence	371 (91.2)	474 (92.6)	845 (91.9)	
	Presence	36 (8.8)	38 (7.4)	74 (8.1)	
	Total	407 (100)	512 (100)	919 (100)	
Perceiver stress n (%)					0.19
	Absence	279 (76.2)	346 (72.2)	625 (74)	
	Presence	87 (23.8)	133 (27.8)	220 (26)	
	Total	366 (100)	479 (100)	845 (100)	
Alcohol consumption n (%)					0.309
	Low-risk	344 (84.7)	432 (84.9)	776 (84.8)	
	Hazardous	52 (12.8)	71 (13.9)	123 (13.4)	
	Moderate-severe	10 (2.5)	6 (1.2)	16 (1.7)	
	Total	406 (100)	509 (100)	915 (100)	
Depressive symptoms n (%)					0.052^#^
	Minimum	362 (89.2)	420 (83)	782 (85.7)	
	Mild	26 (6.4)	50 (9.9)	76 (8.3)	
	Moderate	14 (3.4)	31 (6.1)	45 (4.9)	
	Severe	4 (1)	5 (1)	9 (1)	
	Total	406 (100)	506 (100)	912 (100)	
PA levels n (%)					0.362
	Physically inactive	77 (18.8)	119 (23.1)	196 (21.2)	
	Low active	107 (26.1)	138 (26.8)	245 (26.5)	
	Active	177 (43.2)	204 (39.6)	381 (41.2)	
	Highly active	49 (12)	54 (10.5)	103 (11.1)	
	Total	410 (100)	515 (100)	925 (100)	
Changes in PA patterns n (%)					0.063
	Persistently physically inactive	108 (26.3)	175 (34)	283 (30.6)	
	Became physically inactive	56 (13.7)	74 (14.4)	130 (14.1)	
	Became physically active	76 (18.5)	82 (15.9)	158 (17.1)	
	Persistently physically active	170 (41.5)	184 (35.7)	354 (38.3)	
	Total	410 (100)	515 (100)	925 (100)	
Lower urinary tract symptoms n (%)					0.018†
	Absence/mild	359 (88.2)	429 (83.5)	788 (85.6)	
	Moderate	47 (11.5)	76 (14.8)	123 (13.4)	
	Severe	1 (0.2)	9 (1.8)	10 (1.1)	
	Total	407 (100)	514 (100)	921 (100)	

* Student's t-test

† X^2^ test; # likelihood ratio.

PA: physical activity; SD: standard deviation; BMI: body mass index; HDL: high-density lipoprotein; LDL: low-density lipoprotein; U-RCP: Urinary C-Reactive Protein.

The hierarchical regression, adjusted for demographic, clinical, and lifestyle variables, was used to analyze only the variables associated with statistically significant ED values (Table 3). The results showed that increasing age (each year, OR=1.068; 95%CI=1.048-1.088; p<0.001), high BMI (kg/m^2^, OR=1.041; 95%CI=1.004-1.079; p=0.028), presence of diabetes (OR=1.993; 95%CI=1.158-3.431; p=0.013), and moderate depressive symptoms (OR, 2.052; 95%CI=1.041-4.044; p=0.038) were associated with increased odds of developing ED. In contrast, individuals who became physically active (OR=0.656; 95%CI=0.432-0.980; p=0.041) and those who remained physically active (OR=0.717; 95%CI=0.508-0.992; p=0.045) were associated with decreased odds of developing ED.

**Table 3:** Association of demographic, clinical, and lifestyle factors with erectile dysfunction (n=925)

		OR (95% CI)	p value
Age (each year)†		1.068 (1.049-1.086)	<0.001
BMI (kg/m²)		1.057 (1.022-1.094)	0.001
Age (each year)‡		1.062 (1.043-1.081)	<0.001
BMI (kg/m²)		1.051 (1.015-1.088)	0.005
Diabetes‡		2.024 (1.183-3.464)	0.011
Age (each year)§		1.068 (1.048-1.088)	<0.001
BMI (kg/m²)		1.041 (1.004-1.079)	0.028
Diabetes		1.993 (1.158-3.431)	0.013
Depressive symptoms			
	Mild	1.498 (0.889-2.527)	0.129
	Moderate	2.052 (1.041-4.044)	0.038
	Severe	1.694 (0.439-6.536)	0.444
Changes in PA patterns			
	Became physically inactive	0.768 (0.492-1.201)	0.248
	Became physically active	0.656 (0.432-0.980)	0.041
	Persistently physically active	0.717 (0.508-0.992)	0.045

Hierarchical regression test.

† Adjusted for age (baseline) and body mass index (baseline);

‡ Adjusted for age (baseline), body mass index (baseline), presence of hypertension (baseline), presence of diabetes (baseline), presence of dyslipidemia (baseline), and tobacco use (baseline);

§ Adjusted for age (baseline), body mass index (baseline), presence of hypertension (baseline), presence of diabetes (baseline), presence of dyslipidemia (baseline), tobacco use (baseline), alcohol consumption (baseline), perceived stress (baseline), depressive symptoms (baseline), lower urinary tract symptoms (baseline), and change in physical activity patterns.

OR: odds ratio; 95%CI: 95% confidence interval; PA: physical activity; BMI: body mass index.

## DISCUSSION

This study is the first follow-up analysis to determine the association between self-initiated changes in PA patterns and ED in Brazilian men aged >40 years. Our findings revealed that individuals who became physically active and those who consistently maintained PA had decreased odds of experiencing ED (OR=0.656; 95%CI=0.432-0.980; p=0.041 and OR=0.717; 95%CI=0.508-0.992; p=0.045, respectively). A previous cross-sectional study by Pitta et al. showed an inverse association between PA and ED.^(2,5)^ Our study longitudinally confirmed this relationship using a similar sample. This association persisted after multistep adjustment for age (each year), BMI (kg/m²), chronic diseases, and lifestyle factors that were closely associated with low-grade inflammation.^(6)^ Fernandes et al.^(6)^ also demonstrated that self-initiated changes in PA patterns were linked to low inflammatory levels. Therefore, our results emphasize the importance of adopting and maintaining regular PA patterns to reduce low-grade inflammation, promote cardiovascular protection, and potentially improve ED in adults aged >40 years.

The mechanisms by which PA improves ED are not well defined in the literature. However, the acute response of muscle contraction (induced by PA) results in the production and secretion of several anti-inflammatory proteins (myokines), such as interleukin-6,^(15)^ and increases the bioavailability of nitric oxide levels,^(2)^ which mediate metabolic changes during exercise. The release of interleukin-6 due to muscle contractions increases according to the volume and intensity of activity, leading to an increase in systemic anti-inflammatory cytokines.^(2)^ Additionally, the increased bioavailability of nitric oxide levels from muscle contractions results in acute vasodilation and improved blood and oxygen perfusion, consequently decreasing peripheral vascular resistance.^(2)^ Therefore, chronic PA exposure can lead to low baseline levels of circulating inflammatory markers and improve peripheral vascular resistance. PA can also act as a cofactor for the improvement of other aspects, including anthropometric data such as BMI;^(6)^ laboratory data such as HbA1C; the consequent reduction in insulin resistance;^(16)^ and clinical conditions such as hypertension,^(17)^ diabetes,^(18)^ fatty liver steatosis,^(19)^ and lower urinary tract symptoms^(20)^ Furthermore, PA can have a positive impact on lifestyle factors such as depressive symptoms and stress,^(21)^ alcohol consumption,^(22)^ and smoking,^(23)^ thereby resulting in a decrease in low-grade systemic inflammation, improvement in ED levels, and consequent cardiovascular protection.

This study has both limitations and strengths that must be considered. The strengths include the national cohort of individuals aged ≥40 years, with detailed demographic, health, and lifestyle data collected over 300 and 800 days. To our knowledge, this is the first study to reveal the association between self-initiated changes in PA patterns and ED. This study provided a precise investigation of the variables of interest, particularly PA, after adjusting for several confounding factors in our sample. Trained doctors and health professionals conducted the assessments using validated questionnaires from the literature for variables of interest and confounding factors. In addition, a hierarchical regression model adjusted for clinical, demographic, and lifestyle factors was used to gain a multifactorial understanding of ED in our study population.

Notably, the use of self-report questionnaires instead of objective measures to assess PA is a limitation. However, previous studies^(24)^ have shown that participants’ self-report questionnaires in their everyday lives yielded results similar to those obtained using accelerometers when measuring PA. Additionally, our study may be limited by the lack of information on the dose-response effect of PA on ED, which hinders our understanding of the minimum PA required to reduce ED. The absence of data on certain biochemical variables such as UA and ultrasensitive CRP, along with the small sample size, restricts further interpretation of our initial hypotheses. Finally, the lack of important hormone data, such as testosterone levels, in the assessment of ED could be a potential confounding factor.

## CONCLUSION

In conclusion, this study suggests that in adults and older individuals (aged ≥40 years), making changes to physical activity patterns (becoming physically active) and maintaining these patterns (staying physically active) are important in preventing erectile dysfunction, regardless of age, BMI, and other clinical and lifestyle factors. The association between erectile dysfunction and low-grade inflammation, along with the crucial role of self-initiated and sustained physical activity in preventing erectile dysfunction, has significant clinical implications for the development of strategies and educational programs focused on self-initiated changes in physical activity patterns to reduce the risk of atherosclerotic cardiovascular disease and sexual dysfunction.

## Data Availability

Data are available to reviewers upon request.
